# The third dimension in river restoration: how anthropogenic disturbance changes boundary conditions for ecological mitigation

**DOI:** 10.1038/s41598-020-69796-0

**Published:** 2020-08-04

**Authors:** Martin Guzelj, Christoph Hauer, Gregory Egger

**Affiliations:** 1 Department of Water, Atmosphere and Environment, Institute of Water Management, Hydrology and Hydraulic Engineering, BOKU, University of Natural Resources and Applied Life Sciences Vienna, Vienna, Austria; 2 Christian Doppler Laboratory for Sediment Research and Management, Institute of Water Management, Hydrology and Hydraulic Engineering, BOKU, University of Natural Resources and Applied Life Sciences Vienna, Vienna, Austria; 3 Institute of Geography and Geoecology, Karlsruhe Institute of Technology, Karlsruhe, Germany

**Keywords:** Hydrology, Environmental impact, Riparian ecology, Ecosystem ecology

## Abstract

The goals of the European Water Framework Directive changed the perspective on rivers from human to ecosystem-based river management. After decades of channelizing and damming rivers, restoration projects are applied with more or less successful outcomes. The anthropogenic influence put on rivers can change their physical parameters and result in a different morphological type of river. Using the Ammer River as an example, a comparison between applied systems of corridor determination based on historical maps and data; calculation of regime width; and the change in parameters and river typology are pointed out. The results showed (a) a change in stream power and morphology (b) great difference between the historical and the predicted river type and (c) that regulated rivers can have a near-natural morphology.

## Introduction

More than two thirds of rivers worldwide are highly impacted by human interference^1^.The impact of urbanization on rivers has been described and identified as the “urban stream syndrome”^2^. Some of the main impacts during the last decades are protection and mitigation measures against flooding or land erosion^3–5^. As a result, rivers were channelized and/or straightened to be able to “control” water discharge in case of flooding^1,6^ with the highest priority being the creation of dams and levees^7^. For example, in Germany or in the USA, only about 2[%] of water bodies are still in a natural state^8,9^. Additionally, rivers have been used for water supply; transforming kinetic energy to electrical power^10^; transportation; and drainage of former wetlands for use for agriculture or settlements. All these factors have significant impacts on river morphology and ecological integrity of rivers^10–13^. However, when planning the use of water bodies for human needs ecological impacts were rarely considered^8^.

With the introduction of the European Water Framework Directive (WFD) a need to focus on a broader spectrum of functions of rivers is given as a good ecological status (GES) for rivers^14^ is requested with a basin wide approach^15–17^. As a higher level of water quality is required by 2021 (or 2027 at the latest^15^), restoration projects are taking place all through Europe. Despite EU directives, cultural differences in restoration trends have been observed throughout Europe^18^. Different restoration approaches^19^ had been developed on reach-scale or small-scale basis, and established in the past^19–23^, with most following the theory that habitat heterogeneity leads to a higher biodiversity. However, this theory was rarely a consideration in the short or mid-term perspective^10,24,25^.

According to Shields^26^, small-scale restorations have a higher social acceptance than large-scale restorations, and are more cost effective, while reach-scaled projects show better ecological results related to river rehabilitation. Monitoring showed that not every rehabilitation method is suitable for every morphological type of river^24,26–28^. Measures being successful in the trout region may have not the same effect in lowland rivers. Pretty et al.^24^ pointed out that large scale restoration or habitat heterogeneity can have negative impacts (e.g. increase in bank erosion, change in water depth, incised channels, loss of flat water habitats) on fish habitats and local populations whereas morphologically-suitable measures can lead to an increase in species and populations^23^. Other approaches, such as re-meandering of straightened rivers, showed short-term increase in macro-invertebrates but as the meanders were stabilized with boulders, the morphological effects of erosion and deposition were so limited that this effect faded after a few years^10^. The most sustainable biodiversity increase was derived from large-scale projects where morphological processes have been enhanced, rather than from small-scale, short-term creation of in-stream habitats^29,30^.

Different methods to restore natural diversity in rivers are utilized depending on the aim of the projects (creating habitats for certain species, restoring the historical river pattern to reintroduce historical fauna, etc.)^31^. In most cases these projects attempt to utilise historical status (using two dimensional maps) to determine the space needed. Even though a lot of effort can be put into those projects, including construction work, excavating of former river shapes and beds^26^.

Some projects, such as the restoration of the Skjern river in Denmark^22^, focus on the re-installation of the historical riverbed. In this case, the entire riverbed was reverted to the historical flow path (by excavation). Projects like this require major re-construction and therefore have high costs, and take a long time to recover from construction work to full ecological functionality. Even if outcomes are successful, long-term disruption of the ecological functionality from the construction work need to be considered. Alternatively, this disturbance in the system can also be seen as part of the dynamic change to a river system and can lead to succession processes in vegetation and therefore enhance the entire ecological situation^32^.

In France, a process-based approach has proved to be successful on a larger scale. The “espace de liberté”^33^ is based on the historical range of river transition. In this river restoration approach, a chosen section of the river is given a specific corridor where self-forming processes are responsible for channel formation and turn-overs^34,35^. The concept is based on several parameters, including (1) the historical corridor, which is described by the width of the morphologically active river bed or bandwidth of the meandering sector and (2) the functional corridor width, which is the minimum corridor in which morphological processes can naturally establish. For the functional corridor, restrictions, due to buildings, settlements and infrastructure^35,36^, which have been built in the former riparian area, are subtracted from the historical area and added to the other (non-impacted) side of the river. For the concept of the “espace de liberté”, little anthropogenic mitigation is required, with only space for the self-forming processes requested.

The “espace de liberté” is not the only concept of its kind, nor is it the oldest in application. There are similar approaches in setting up corridors for river transitions throughout the world, including in northern India (which was already described in 1903^37^), in the USA^38^ and in Europe^39^. In most of these cases, the river catchment and reaches were previously undisturbed. This implies that the river has retained basically the same morphological type for the last few decades.

As mentioned before, only about 2[%] of rivers in Germany are still in a “natural” condition. Therefore, approximately 98[%] are impacted or strongly affected by human alteration such as straightening, lowering of the riverbed, alteration of banks (in meander bends) and other factors. One question arising is the impact of anthropogenic pressures on riverine landscapes and floodplains. For example the Tagliamento River in northern Italy shows a local difference in morphological patterns where pressure is applied to the riverbed by gravel mining and a forced narrowing by levees^39^.

The classifications of morphological river patterns^40^ are based on characteristic physical parameters including e.g. (1) channel width and depth, (2) flow velocity, (3) slope and parameters concerning, (4) bed material and (5) sediment load. If one or more of these parameters change, the river morphology response will change as well.

Various studies regarding river restoration projects have assessed that most restoration projects throughout the world have had little success (or have failed) due to too little understanding of the geomorphological and hydraulic processes, of changed conditions in riverbeds or discharge patterns, or of ecological interaction between biota-fauna and the mentioned processes^19^. Even though maps can be used to determine both the width of the riparian area and historical river types, very little information about the parameters used for river morphology is given by these^26,41^. Maps usually show only the two-dimensional spatial location of the riverbed, but lack information about grain size, erodible material, changes in slope or channel discharge capacity^42^.

There has been a call for scientific assessment of project areas that shows the current state of the site and helps practitioners to choose the right approach based on the actual situation on site rather than on historical approaches^27,30,43–45^. However, this has not yet been implemented in a reliable or practical way. Thus based on the lack of knowledge regarding various concepts in river restoration, the following research question has been formulated for this article:

How important are changes in the physical variables of high flows (e.g. flow velocity, stream power, bed shear stress) due to channel modification for a successful prediction and implementation of river restoration concepts? As side-effects the uncertainties in state-of-the-art concepts for corridor determination were highlighted and targets for future research suggested.

The research questions were tested at the Ammer River/Germany. At this river (1) historical data is well known, and (2) significant human alterations are evidenced along the river section. This makes the river an interesting case study to test both research questions. Different restoration concepts like the “espace de liberté”^33^ and development corridor^46^ were applied, in which the required hydraulic parameters for flood dynamics were derived by hydrodynamic-numerical modelling. For the determination of the river type, remaining oxbows and the actual riverbed were measured and compared to see if there is a difference in parameters^47,48^.

### Site description

**Figure 1:**
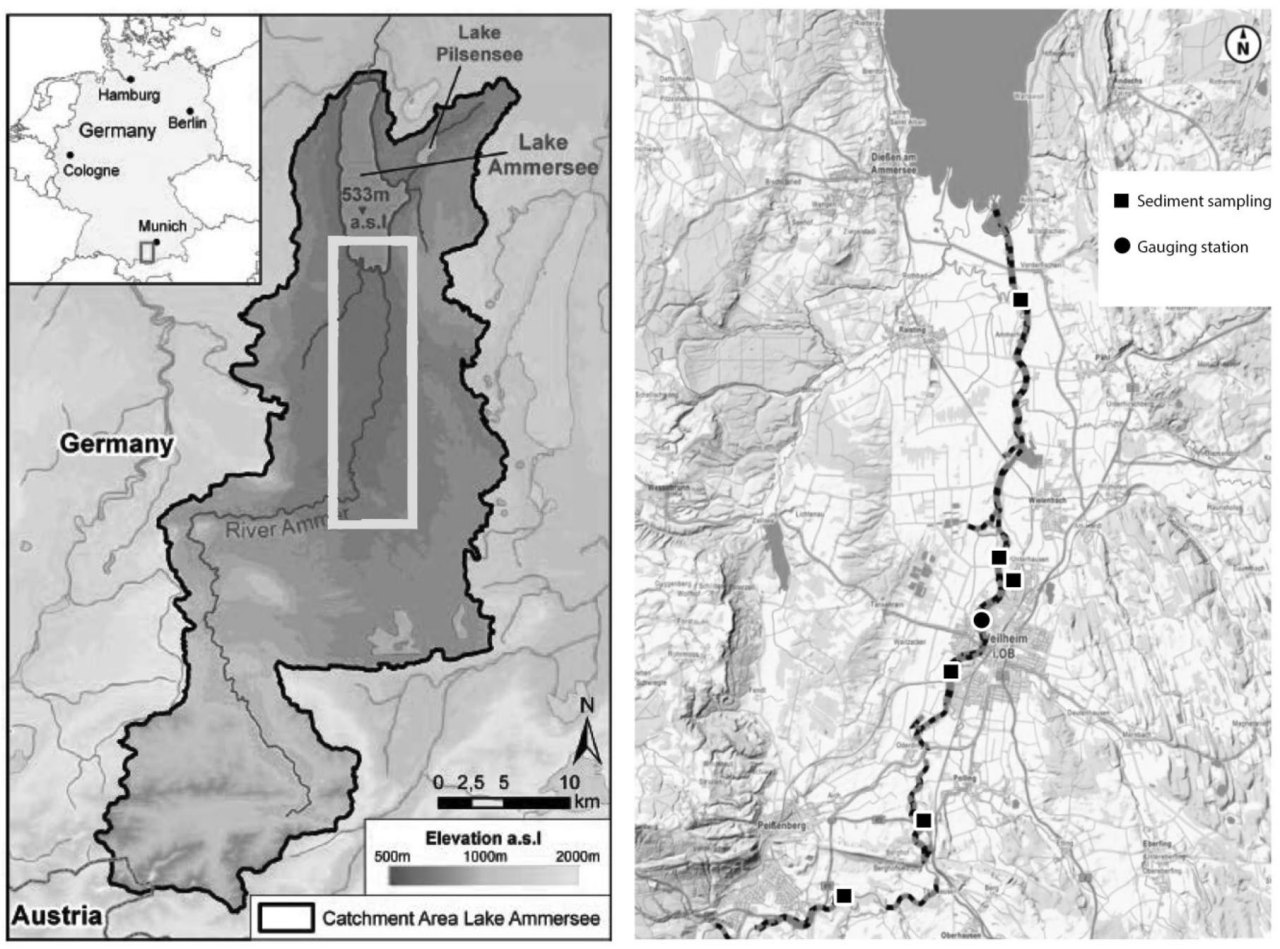
Location of the Ammer River catchment with research area marked and the channelized river-section indicated by levees along the research section on Ammer River (Sources:^57^, edited with ArcMap 10.4^58^).

The Ammer River drains a catchment of 600[km^2^]^49^. Starting as Linder at the border between Austria and Germany (47°33′50″N 10°56′10″E) the river seeps into the karst and surfaces southeast of Oberammergau as Ammer springs. Over a length of 80[km], the Ammer River crosses various zones of the limestone Alps and the northern alpine foodhills before it flows into Lake Ammersee^49^. The research area is situated between Peißenberg (47°46′34.2″N 11°03′15.5″E) at the end of the Ammer Gorge and the Ammer Delta (47°56′52.2″N 11°08′02.0″E) (Fig. 1).

Based in the middle of the South German Molasse Basin (SGMB)^50^, the Lake Ammersee basin was formed by glacial movement. The main waterbody in this area is the name-giving Lake Ammersee, which is a pre-alpine lake. The Ammer River is the main river in the investigated catchment. It has alpine characteristics, with high amounts of sediments. The lake was already partially naturally filled by gravel, sand and clay as alluvial parts, as well as lake sediments and turf^51–55^. The Ammer valley is dominated by kalkpaternia soiltype on top of limestone sands and gravel. According to the Bavarian Institute for Agriculture, the gravel layer starts at about 0.5 to 1[m] depth^56^. According to the data provided by Copernicus Land Monitoring Service (CLMS) the research area is dominantly of agricultural land use (grassland 50.1[%], forest areas 23.1[%] and cropland 13.7[%]) and urban areas (13.0[%]).

The nival and sub-alpine characteristic of the runoff regime is described as mainly rainfall-induced high flood events in summer, and low water levels in winter (see Table 1)^46^. The permanent gauging station of Weilheim^59^ is situated in the section chosen for the analysis. The mean daily discharge records of the station go back to 1925^60^. The average discharge and the annuity values derived from the WWA Weilheim are presented in Table 1.

**Table 1:** Average discharge and calculated flood annuities with correlating discharge values for the gauging station Weilheim for the period from 1925 to 2018.

Gauging station Weilheim	Discharge values	HQ_T_
	NQ = 26 m^3^ s^−1^	HQ_1_ = 120 m^3^ s^−1^
	MNQ = 56 m^3^ s^−1^	HQ_2_ = 150 m^3^ s^−1^
	MQ = 153 m^3^ s^−1^	HQ_5_ = 205 m^3^ s^−1^
	MHQ = 164 m^3^ s^−1^	HQ_10_ = 255 m^3^ s^−1^
	HQ_max_ = 649 m^3^ s^−1^	HQ_20_ = 315 m^3^ s^−1^
		HQ_50_ = 410 m^3^ s^−1^
		HQ_100_ = 480 m^3^ s^−1^
		HQ_1000_ = 750 m^3^ s^−1^

*NQ* low flow discharge, *MNQ* mean low flow discharge, *MQ* mean discharge, *MHQ* mean highflow event, *HQ*_*max*_ maximum discharge^60^.

The first recorded modifications (e.g. outlets for mills) in the Ammer catchment took place in the early sixteenth century. The first morphological modifications of river bends were undertaken in mid-nineteenth century to ease the transport of wood logs. However, the main engineering measures took place between 1920 and 1937, e.g. straightening and the construction of levees. Between 1920 and 1924, the river mouth into Lake Ammersee was “reconstructed” as part of the straightening program for flood protection. At the time, the location was changed from the western part of the bay (47°56′24.2″N 11°07′05.6″E) to the village of Fischen (47°56′13.9″N 11°08′31.7″E). The main goal of the construction work in the late 1930s was to create work for the people and was not really based on flood control issues. This had special impact on the flood protection scheme upstream of Weilheim. The entire flow path between the city of Weilheim and Lake Ammersee was reduced from 25[km] to about 13[km] by channel rectification. Alongside the main channel, the swamp and turf areas were drained to gain more agricultural land and to be able to harvest the turf. The river bed was lowered to ensure drainage throughout the year^49^.

According to the GIS platform of the Bavarian Ministry for Digitalisation, Broadband and Surveying, and the WWA Weilheim, almost the whole section is channelized with levees^57^ (as shown in Fig. 1). The levees are part of the applied flood protection scheme which is constructed to the extension of HQ_300_ highflood event according to the WWA Weilheim.

## Methodology

The methods applied in this study are based on: (1) analysis of historical maps to derive the former riparian area, followed by (2) analyses of the physical parameters described earlier. Additionally, (3) hydrodynamic-numerical modelling has been used to assess changes of the in-stream parameters such as stream power, flow depth and bankfull discharge. This was later compared to the historical channel characteristics and the straightened, regulated river section. As a final step (4) the morphological type of the river was determined using DaSilva’s and Jäggi’s classification based on the data derived from the comparison.

### Analysis of historical corridors

**Figure 2:**
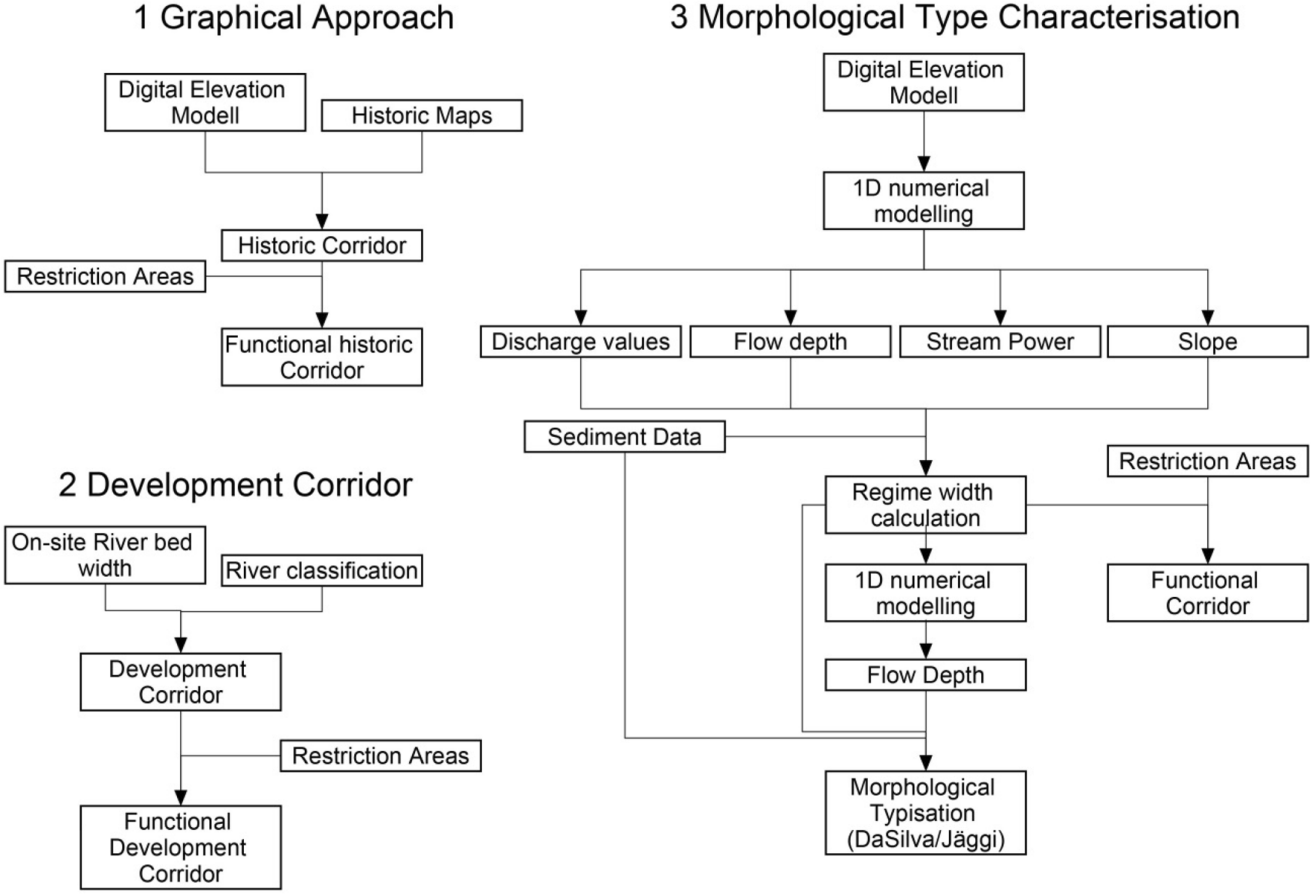
Work flow diagram with input data needed for (1) graphical approaches (historical corridor and historical maps used as basis) (2) development corridor approach (riverbed width and classification of rivers^53^) and (3) the 3 dimensional approach with morphological type characterization.

The aim of the presented study was to investigate the range of complexity needed for the determination of river corridors in terms of restoration based on self-forming processes. The study increases in complexity of analysis from the “traditional” analysis of historical maps, to methods, which included the “third-dimension” of channel characterisation, including hydrodynamic-numerical modelling (Fig. 2). In the following section, the methods of the determination of the various corridors (graphical and calculated) are presented. First, (1) the graphical approach has been used as defined in the concept of the “espace de liberté” suggested to define the historical corridor, while (2) the calculated development corridor from governmental suggestions for practitioners has been used to derive the functional corridor. Next (3) the hydraulic-geometry-based approaches were used to include the third-dimension for the first time in the analysis to determine first and second order erosional corridors, and finally (4) channel patterns are predicted based on the results of (3), which may evolve due to the erosional width of the first and second order channel changes.

### Graphical analysis: two dimensional approach

**Figure 3:**
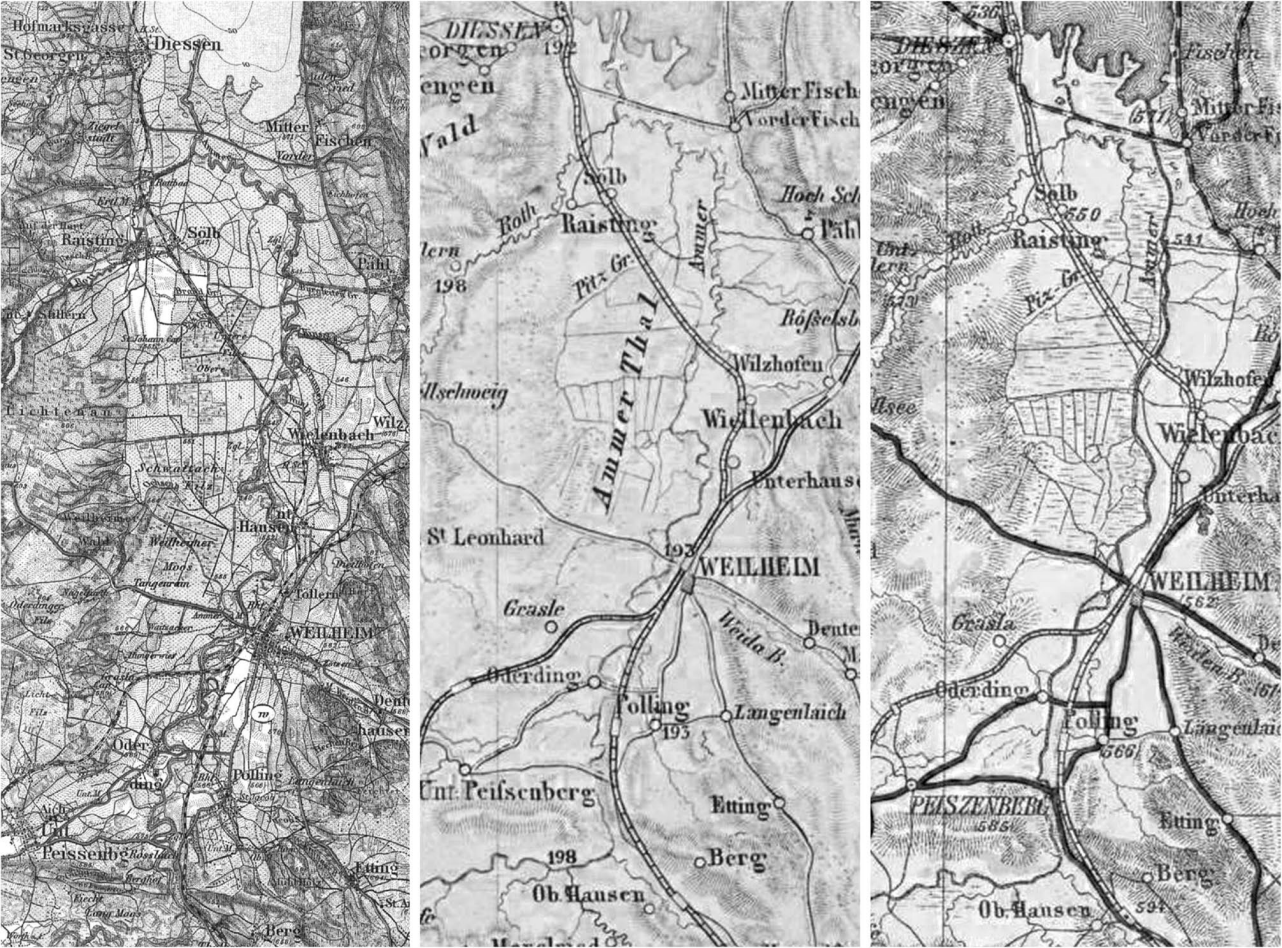
Historical maps of Ammer region 1902^61^, 1930^62^, 1941^63^ (12.11.2019) used for determination of the historical shift of the riverbed showing the alteration by human interference due to regulation and flood protection schemes.

The concept of “espace de liberté” is based on the idea that the river should restore itself with very little human interference within the limits of the historically active channel and floodplains (compare Fig. 2)^35^. To address the questions mentioned before, historical maps of the river section between Peißenberg and the river mouth into Lake Ammersee have been gathered and analysed. Maps available from the State Library of Bavaria^61^ were used to assess the flowpath of the historical Ammer River. The maps were geo-referenced with ArcGIS on a multipoint basis to each other in a first step and later on with the actual digital elevation model (DEM). To be able to reference the maps, intersections on roads as well as landmarks and building structures were taken into account. Special focus was set on maps in which the river was shown in a way which represents the morphological patterns along its flow path. With this restriction, the resulting maps chosen for further analysis (Fig. 3) were from the years (1) 1842^61^ as the earliest map, (2) 1902^61^ as the map before the first straightening, (3) 1930^62^ and (4) 1941^63^ as last historical map due to the highly changed flow path. Examples of the maps are shown in Fig. 3. The centreline of the historical river path was used in combination with the measured width of the remaining undisturbed oxbows. The centrelines were buffered in ArcGIS and the outer parts of the river bends connected with a polygon. Those maps are crucial for the definition of the historic morphological corridor C_hist_ which defines the maximum extension for the “espace de liberté”, the former river shape and the morphological processes. It also is an indicator of the riparian area which previously surrounded the river.

### Developing corridor: two dimensional approach

In 2014, the German Ministry for Environment published a guideline (as part of the WFD goals), in which each river in Germany is assigned into one of 21 classes^53^. For each class, typical characteristics, morphological shapes and cross-sectional appearance of a natural state river are explained and defined. According to Dahm et al.^46^ the Ammer River is part of the Limestone Alps rivers. For the minimum and maximum developing corridor (*W*_*dev_min*_ and W_dev_max_) the average constructed riverbed width W_constr_ had to be calculated by using geometrical data from observations of current anthropogenically-modified status (Fig. 2). According to the advice of the German Ministry for Environment, for rivers in the limestone alps region^46^ the single steps are:

1 $${W}_{nat\_pot}={5W}_{constr}$$

2 $${W}_{{dev}\_{min}}=2{W}_{{nat}\_{pot}}$$

3 $${W}_{{dev}\_{max}}=5{W}_{{nat}_{pot}}$$

whereas W_nat_pot_ stands for the potential natural riverbed width which is calculated from W_constr_. Charrier (2012) suggests to use ten times the bankfull width as an equilibrium width for the “espace de liberté”, which equals W_dev_min_^64^. This calculated width was applied as a buffer zone in ArcGIS to the actual river line to define the corridors.

By overlaying the (1) historical corridor, (2) the actual waterbody (including main channel and remaining oxbows) and the W_dev_min_ and W_dev_max_ corridors, the functional corridor is defined as the intersected area. Going on from the resulting polygon, industrial and settlement areas as well as infrastructure were subtracted in ArcGIS and the resulting area is the theoretical corridor the river would need for its natural development.

### Hydraulic: geometry equations: three dimensional approach

Deriving from the regime theory, which is based on the idea that a morphologically active flood erodes in all directions up to a natural equilibrium width, various authors set up empiric formulas to calculate the final extension of the riverbed (Fig. 2). For the presented study it was necessary to apply these formulas from opening up a regulated channel to the status of the full development of a riverbed which can be divided into primary and secondary erosion. The later explained formulas after Yalin (1992) and Ashmore (2001) were chosen due to the fact that the parameters for the setup best suit the existing data from field research. Values used for calculating derived from: (1) flood statistics for HQ_1_; (2) the numerical model for the bank full discharge HQ_bf_ and the slope J; and (3) sampling data for the median diameter of the top layer of the bed material.

#### Corridor of primary erosion

After the removal of stabilizing elements, the river channel widens but remains “straight” meaning the morphological type of river remains. This process is known as primary erosion. The main factors are the discharge (Q) and the grain size (e.g. d_50_). The resulting width is the equilibrium width (b_eql_) in meters^65^.

4 $${b}_{eql}=1.42\cdot \sqrt{\frac{Q}{{u}_{*}}}=1.5\cdot \frac{{Q}^{0.5}}{{d}_{m}^{0.25}}$$

Ackers and Charlton^66^ discovered that depending on the discharge, rivers with lesser slopes (J < 0.3%) have a higher tendency to stop side erosion than rivers with greater slopes (J > 0.5%).

#### Corridor for secondary erosion

As soon as the process of primary erosion has reached its maximum width, secondary erosion processes may occur. These processes are responsible for the formation of riverbed structures (ripples, banks, etc.) as well as for changes in the river profile (e.g. braiding, shifting islands). In literature, different formula can be found^67–71^. For this study, the formula after Ashmore (2001) was chosen due to the best accordance for the empirical setup of the formula and the onsite data.

5 $${b}_{eq, sec}=0.0098\cdot {\left(\rho \cdot g\cdot Q\cdot J\right)}^{0.7777}\cdot {d}_{50}^{-0.7}$$

with ρ [kg m^−3^] is the water density, g [m s^−2^] is gravity and Q [m^3^ s^−1^] equals the morphological active discharge (HQ_1_, HQ_2_).

The result shows the width of the eroded corridor and therefore the morphological active channel. This width has been used as input for the morphological type characterisation.

### Morphological type characterisation: three dimensional approach

It was necessary to evaluate if present boundary conditions of channel formation fit with the historical channel characteristics. Bathymetric data have been collected for the years 2004 and 2009. These data of the regulated river were further compared to the historical (unregulated) situation by measuring the bathymetry of the historic river bed (see Fig. 3) derived from remaining oxbows. Therefore, irregular distanced cross-sections (n = 7,384 including interpolated cross-sections) were placed in the Digital Elevation Model (DEM) and were cut through the channelized river section and the remaining oxbows along the research area. The average degradation for 3 sections was determined by measuring the height difference between the lowest points. The 3 sections are geographically based: (1) Peißenberg (47°46′45.6″N 11°04′27.9″E), (2) Unterhausen (47°51′44.8″N 11°08′32.1″E), and (3) Vorderfischen at the outlet to the former river bed (47°55′30.7″N 11°08′44.9″E). The data from the DEM (derived from the high flood model) had been edited from LIDAR data, but did not reflect the historical situation in an adequate way. At the sections of Unterhausen (47°51′36.5″N 11°08′45.6″E) and around Pähl (47°91′35.1″N 11°14′97.9″E), additional cross sections (n = 23) were gathered with nine cross-sections in the first section and 14 along three oxbows further downstream. This was necessary as the oxbows had been prone to siltation over the last decades due to material input from small tributaries^72^. To be able to determine the historical slope and other relevant parameters, it was important that the data gathered reflect the situation before the river regulation. Therefore the points collected were measured from the former gravel bed though the layers of accumulated fine materials^73,74^. The siltation layers were not part of the research for this paper.

In a second step (Fig. 2), and for the definition of the morphological channel types, the measured and predicted parameters were analysed according to Jäggi (1983) and DaSilva (1991) to be able to compare the historical river type and resulting river pattern due to the onsite measured parameters resulting in X, Y, Z and the slope J. For the regime width, a substitutional cross-section was calculated by linear extrapolation:

6 $$X=\frac{b}{{d}_{m}}$$

7 $$Y=\frac{b}{h}$$

8 $$Z=\frac{h}{{d}_{m}}$$

where b represents the water surface width and h is the flow depth.

**Figure 4:**
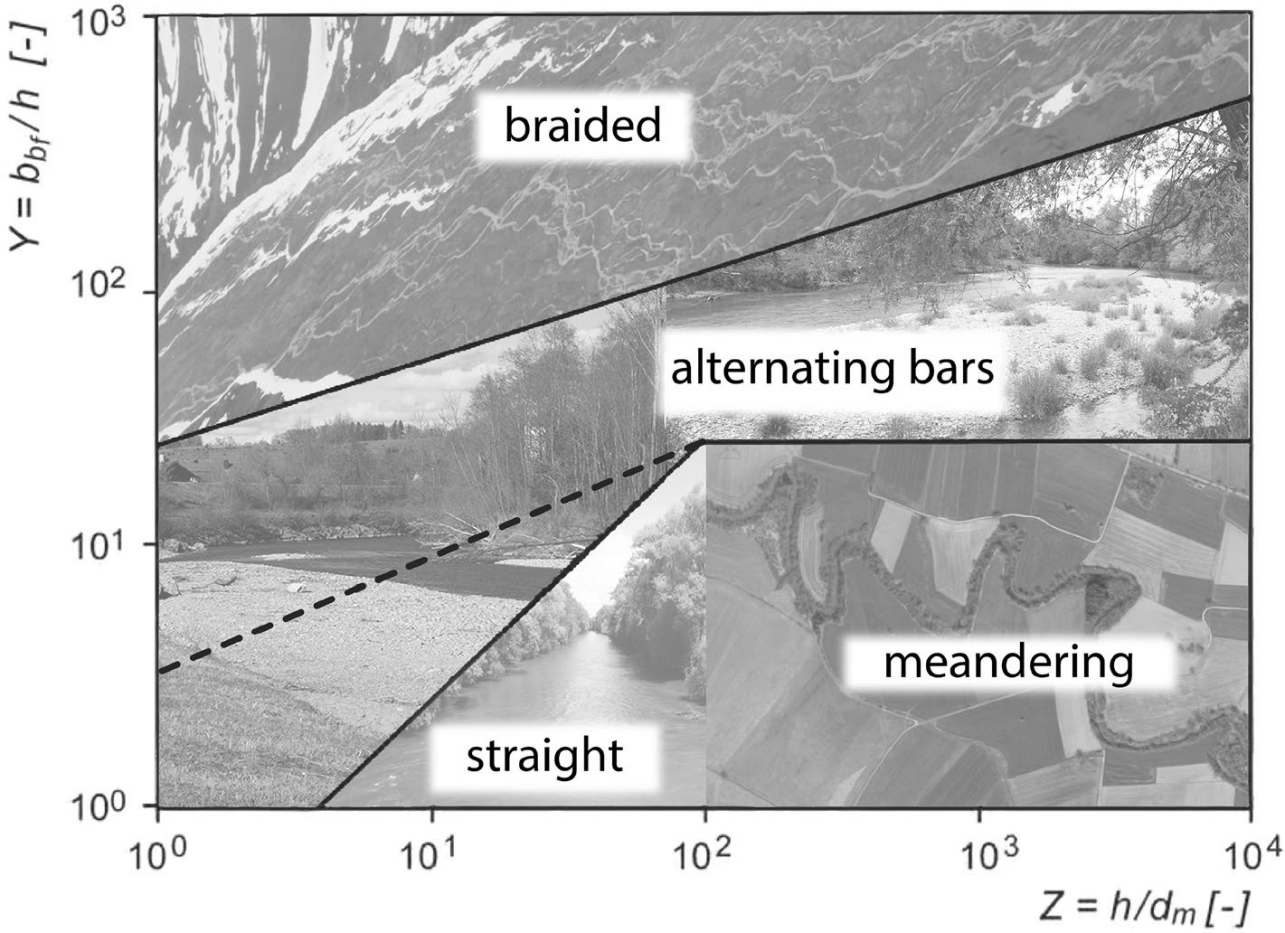
Modified morphological river pattern diagram after DaSilva (1991) and Zarn for determining river characterization on behalf of discharge, flow depth and the width of the wetted area (compare^75^).

**Figure 5:**
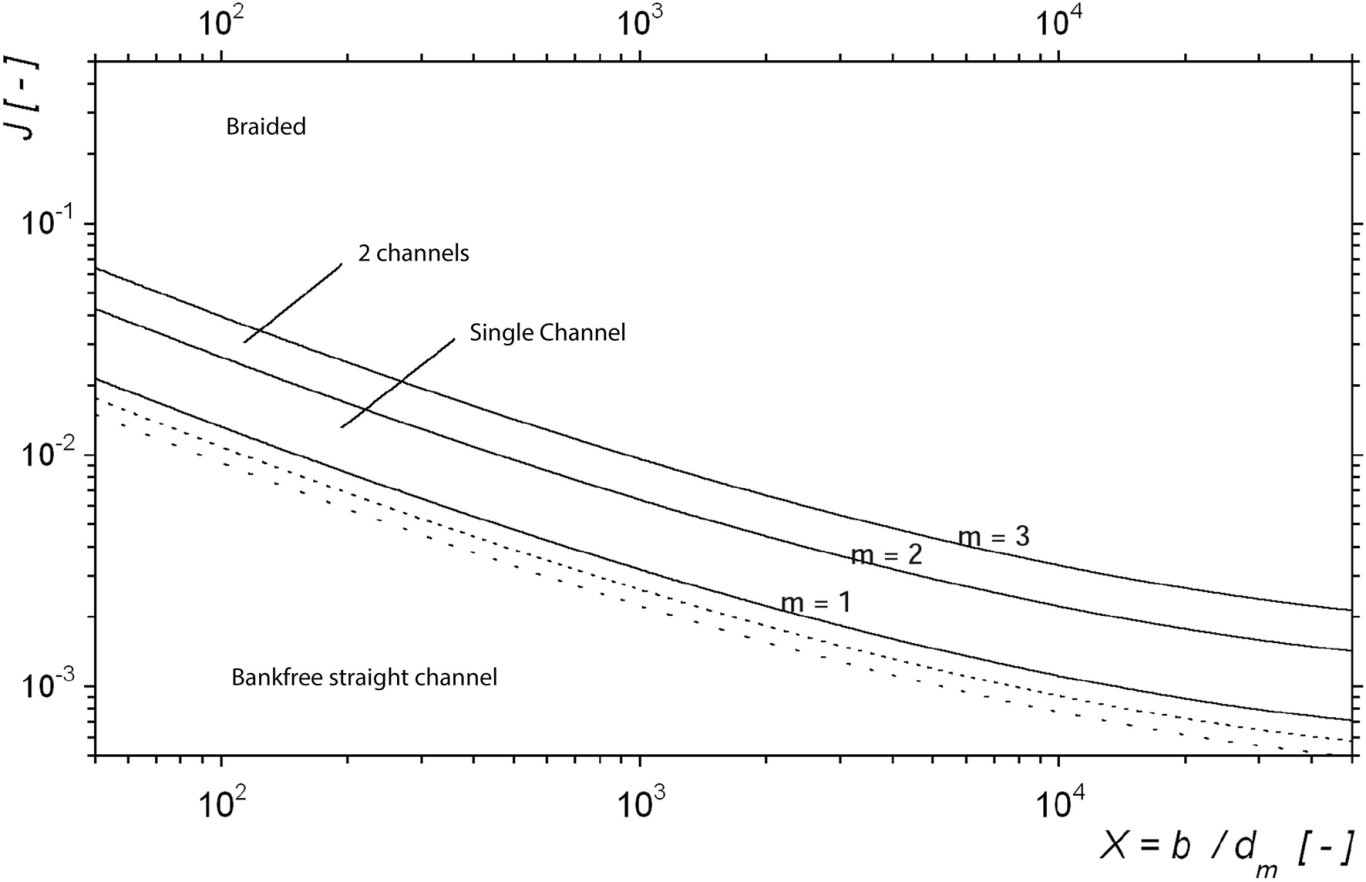
Diagram after Jäggi (1983) for the determination of river types between straight river, single channel and multi-channel rivers^47^.

The correlation between X, Y, Z and J, and the resulting morphological type of rivers are shown in Figs. 4 and 5. While the definition after Zarn and DaSilva was used to define the pattern with the current parameters, Jäggi’s diagram was used to analyse the resulting pattern in more detail. This diagram defines the threshold level between a straight river with no banks, single channel river with alternating banks, and a multi-channel or braided river as shown in the graph (Fig. 5). According to Jäggi (1983) there is a correlation between the slope J, the grain size, and the width of the wetted area (X)^47^.

### Hydraulic modelling

Hydrodynamic-numerical modelling was applied for the characterisation of channel development (approaches 3 and 4) and for analysing the impact of hydro-morphological parameters on river forming processes for the selected river section. Here, a one-dimensional hydrodynamic-numerical model was applied. One-dimensional interpretation of governing equations has found a widespread application in hydraulic and environmental engineering^76,77^. The main equations on which 1D models are based are the principles of (1) mass balance (Eq. 9) and (2) the balance of momentum continuity (Eq. 10)^78,79^.

9 $$\frac{{\delta A_{T} }}{\delta t} + \frac{\delta Q}{{\delta x}} - q_{l} = 0$$

where Q_(x,t)_ = discharge [m^3^ s^−1^], A_T_ = cross-sectional area [m^2^], q_l_ = lateral inflow per unit length [m^2^ s^−1^].

10 $$\frac{\delta Q}{{\delta t}} + \frac{\delta QV}{{\delta x}} + gA\left( {\frac{\delta z}{{\delta x}} + S_{f} } \right) = 0$$

where S_f_ = friction slope [−], z = water surface [m] (equal to z_0_ + h (water depth of control volume)), V = control volume [m^3^], g = gravitational acceleration [ms^−2^].

For the analysis, the one-dimensional unsteady flow model HEC-RAS was used. The model was chosen because of its capabilities for sub-/supercritical modelling^80^ and multifunctional parameter analysis for channel flow. The modelling package HEC-RAS uses the 1D St. Venant equation to calculate open channel flow, based on a four-point implicit finite difference scheme, allowing modelling over larger time steps than explicit numerical schemes 80. For the applied one-dimensional approach, phenomena such as the Coriolis force have been neglected.

Using software bankfull discharge capacity [m^3^ s^−1^], flow velocity [ms^−1^], the specific stream power [Nm^−1^ s^−1^], as well as the slope of the riverbed [-] on the reach-scale were calculated. For modelling, a representative section close to Unterhausen was chosen (47°51′33.2″N 11°08′31.1″E upstream–47°51′48.8″N 11°08′34.6″E downstream) including both, the historical bathymetry and the regulated existing one.

### Bed material

Grain size distribution is one of the controlling factors in river morphology^81^. Samples to determine the grain size distribution were collected at four stations (Peißenberg, Oderding, Unterhausen, Vorderfischen) and one sample was taken out of the oxbow at Unterhausen. To receive representative data, the material was collected in a low water period in early spring (2017). On all sections, a surface layer and a sub-surface layer sample was collected. The samples were taken from gravel bars in the river and analysed at the lab of the University for Life Science, Vienna. The resulting grain size distributions were used to determine the d_50_ or d_m_, which is used for the calculations as well for the corridors as the river type pattern. In combination with the samples of bed material taken in each of the modelling sections, the actual situation on the river can be properly described. The grain size distribution of the samples and the hydraulic characterization by various parameters in the section were also the basis for the determination of the evolving river type.

### Hydrology

The gauging station of Weilheim is situated within the research area. The discharge has been recorded since 1925. To define the current situation and to analyse possible trends in discharge, the available dataset (1926–2017) was taken into account for the hydrological situation. The method of the cumulative deviation of the mean was applied to compare single years and seasons^82^. The average of the whole time series as a constant (*c*) is subtracted from each value (x_j_). The results are accumulated for the timescale to be analysed (seasonal, yearly or for decades) and plotted on the original time scale^82^.

11 $${S}_{i}=\sum_{j=1}^{i}({x}_{j}-{c})$$

Based on mean daily discharge values, analyses were performed for the yearly period as well as for the seasonal discharge behaviour. The results of the analysis were used to investigate possible trends for the future extrapolation of discharge patterns for the evolving river morphology in the simulation.

The discharge values applied to the hydrodynamic-numerical model are the main characteristic discharges (1) NQ, (2) MQ, (3) HQ_1_ (Table 1) and (4) bankfull discharge, which has also to be determined from the HN-model in combination with the data collected from the oxbow (historic situation). The bankfull capacity for the main channel has been defined at the height of the surrounding overbank areas and not the full capacity of the regulated section with levees.

## Results

### Analysis of historical corridor

Changes of the Ammer River and the morphological constraint due to anthropogenic interference over the decades are shown in Fig. 3a. Displayed in the map are two main stages of river history—which are: (1) 1,840 river course as a “near natural” state and (2) 1937 after the main construction work was done and the river had its present shape. Our analysis enabled us to identify three river sections with different characteristics. Stretch one is from Peißenberg to Polling. Here a relatively straight, smoothly bending river form can be seen. For the next section downstream from Polling to Wielenbach, the river shows a pattern of meandering/bending with stretched meanders. Further downstream the meandering type dominates, with a high winding rate along the flow path to the river mouth.

**Figure 6:**
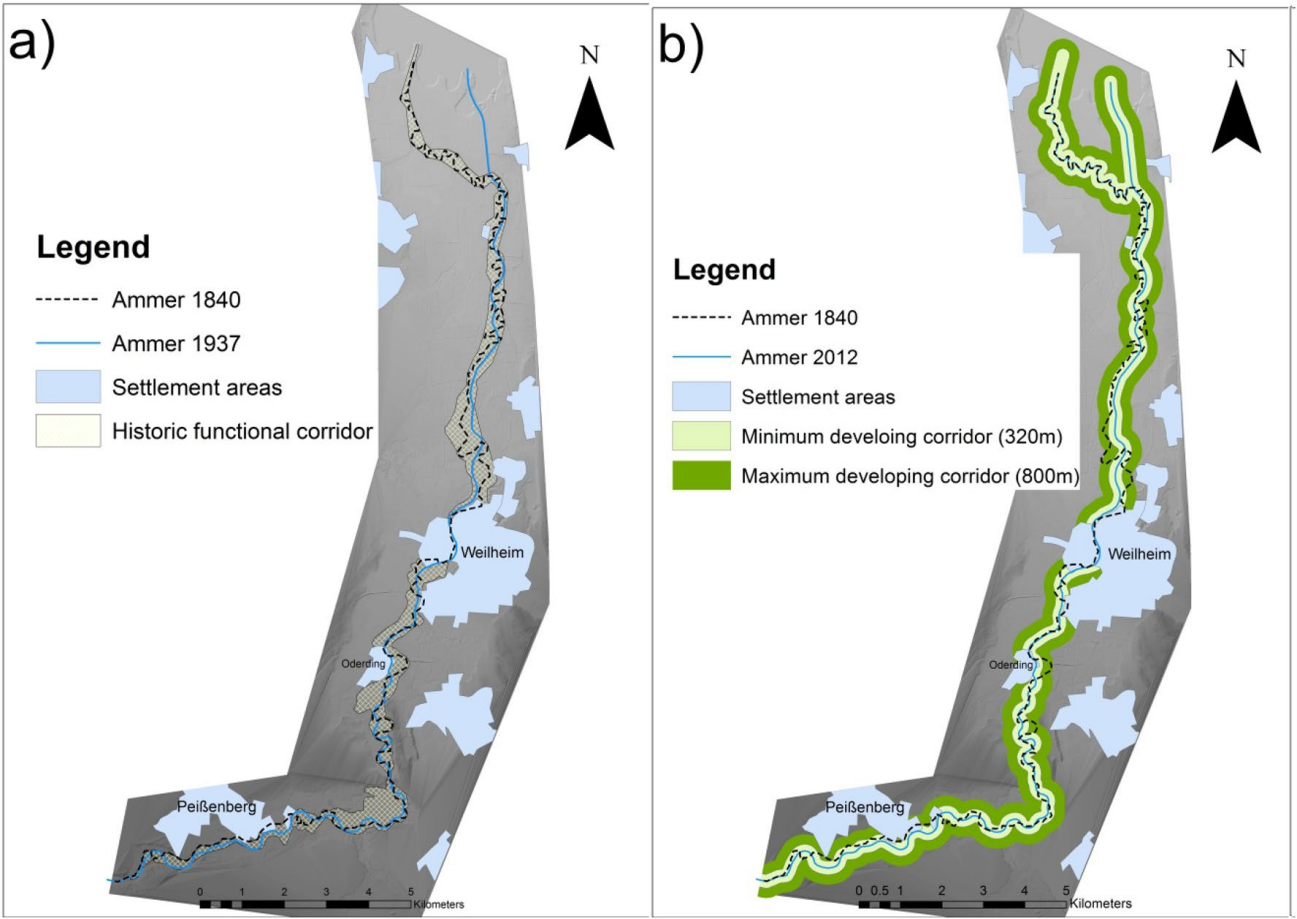
(**a**) Displaying the two main steps in changes of the river’s course (1840/1937) and the historical corridor; (**b**) functional developing corridors (min. 320 m/max. 800 m) calculated after Dahm et. al.^46^ for the river bed of 2012 with settlement areas as restrictions. The winding part, found in both pictures, is the historic flow-path while the corrected path is rather straight. The maps were created with ArcMap 10.4 (https://www.esri.de/landingpages/arcgis-10-4).

The 1937 river course is similar to the 1,840 path for the first section (Peißenberg to Polling). Here the riverbed remained mostly unchanged and seems to be stabilized. Further downstream, all bends were cut off and the river became a mostly-straight smoothly winding river along the old river bed. The final change can be seen at Fischen, where the river channel has been constructed straight into the lake. As result of the historical analysis, the corridor definition can be seen as the simplest form of determining boundaries for self-forming channel development. The corridor was defined by the 1,840 river path and the remaining oxbows. While being relatively narrow for the first section (150–200 m), it widens to its maximum width around Weilheim (750–850 m) and narrows again at the beginning of the strongly winding part (200–250 m). As shown in Fig. 6a, the new entrance into the lake is completely out of this corridor. It can be seen that even though it is in an almost natural state, the river corridors vary by a factor of about 3 to 4 and at least 3 sections have to be defined.

### Developing corridor: two dimensional approach

The basis of the developing corridor (after Dahm et. al.^46^) is the constructed riverbed width W_constr_. The result of the analysis of 15 cross sections along the whole research area derived from the 1D hydrodynamic-numerical model showed an average value of W_constr_ = 32[m] (Standard Deviation = 39[m]) between the levees. Following the formula (Eqs. 1–3) the three buffering zones are: (1) W_nat_pot_ = 160[m]; (2) W_dev_min_ = 320[m]; and (3) W_dev_max_ = 800[m]. As can be seen in Fig. 6b, main restrictions are found at the areas of Peißenberg, Oderding and Weilheim, with the highest impact given by settlement areas on both sides of the river.

### Hydraulic: geometry equations: three dimensional approach

Assuming a natural discharge capacity of around the annual flood event HQ_1_ = 120 [m^3^ s^−1^] as the in-bed structure forming flood event has been chosen. For the main channel, the bankfull discharge was set to HQ_bf_ = 280[m^3^ s^−1^], the simulation with the historic riverbed showed a maximum discharge of HQ_bf_hist_ = 125[m^3^ s^−1^] and therefore this value has been used. The remaining input values for the modelling and analysis of the main channel are described in Table 2.

**Table 2:** Input data for the calculation of the regime theory width derived from statistics (yearly flood event discharge (HQ_1_)); modeling approach (Bankfull Discharge (HQ_bf_) and the slope J) and field samples (Sediment diameter (D)).

HQ_1_ [m^3^ s^−1^]	HQ_bf_ [m^3^ s^−1^]	HQ_bf_hist_ [m^3^ s^−1^]	J_Unterh_ [–]	J_Peißenb_ [–]	J_hist_ [–]	D_50Unterh_ [m]	D_50Peißenb_ [m]	D_50hist_ [m]
120	280	125	0.00303	0.00335	0.00051	0.012	0.078	0.024

The results show that the slopes vary only by 0.32[‰] comparing the channel parameters between Peißenberg and Weilheim. The d_50_ decreases from 78 to 12[mm] in downstream direction (Fig. 2). However, the slope of the historic riverbed in the area of Unterhausen steepened by about 600[%]. This increase of gradient is partly due to the correction of the river, and partly due to excavation to drain the land. A direct effect on the riverbed change due to increased shear stress as following up effect with this increase cannot be reported. This might be due to weirs and similar constructions.

Calculating the corridors for the assumed historical bed forming discharge (HQ_1_) and for the maximum morphologically active discharge (Q_bf_), the resulting corridors for the primary erosion at Peißenberg are 31[m] for b_HQ1_ and 47.5[m] for the bankfull discharge (b_HQbf_). At Unterhausen, further downstream, the b_HQ1_ is calculated 49.6[m] and b_HQbf_ has a predicted value of 75.8[m]. It can be seen that the variation in discharge by the factor of f_discharge_ = 2.3 leads to increase of the width by the factor of f_width_Q_ = 1.53 for both sections. The change in slope (f_slope_ = 1.1) and bed material from 0.078[m] to 0.012[m] (f_sediment_ = 6.5) results in a wider corridor by the factor of f_width_bed_ = 1.6 at the same discharge.

For the resulting width of the channel (b_eq,sec_) after the river type forming processes, the formula for secondary erosion after Ashmore (2001) was used. The same discharges as for the primary processes were applied. Using Eq. (5) for the upstream section at Peißenberg, the resulting widths are b_eq,secHQ1_ = 36.4[m] and b_eq,secHQbf_ = 70.3[m] whereas for the part downstream of Weilheim b_eq,secHQ1_ = 124.7[m] and b_eq,secHQbf_ = 241[m] are calculated. For the secondary equilibrium width, results showed that the influence given by bed material and slope is much higher than the influence given through the change in discharge. The discharge factor changes to f_width_Q_ = 1.9 and the factor for riverbed parameters to f_width_bed_ = 3.4. The comparison between the initial erosion and the result shows that the maximum difference between b_HQbf_ and b_eq,secHQbf_ can be found in Unterhausen with a predicted Δb_Qbf_ = 165.2[m], while for the Peißenberg it is Δb_Qbf_ = 22.8[m]. A similar result can be seen for Δb_HQ1_ with its maximum value of Δb_HQ1_ = 75.1[m] in Unterhausen and the lower value of Δb_HQ1_ = 5.4[m] at Peißenberg.

### Morphological type characterisation: three dimensional approach

**Figure 7:**
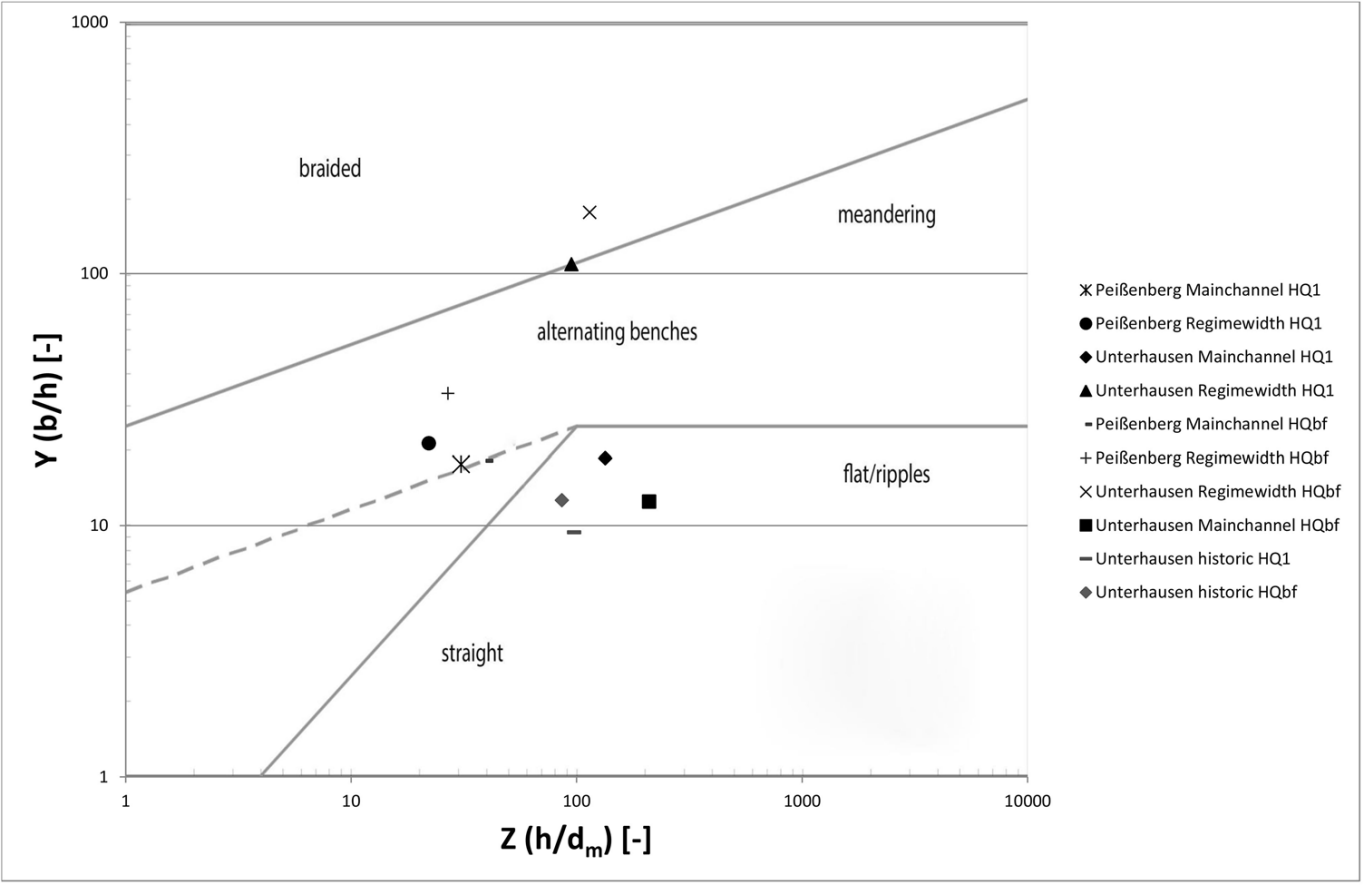
DaSilva-Diagram including the results for Peißenberg and Unterhausen main channel, and equilibrium width, historical.

The morphological results can be divided into three pattern clusters as there are: (1) straight channel/slightly meandering, (2) alternating bars and (3) braided river (Fig. 7). In cluster (1), four river stretches with similar behaviour can be found. All of them are results of the morphological typisation at the area of Unterhausen. Two of them describe the on-site situation, while the other two result from the analysis of the historical pattern. This means that without any changes in cross-section (such as widening), this would be the river’s “natural” form with the given boundary conditions. Thus, the Ammer River would be a straight river in this area with the parameters of today. For the historic riverbed, a straight to meandering river form is predicted. The resulting parameters X, Y and Z can be seen in Table 3 at the end of this chapter.

**Table 3:** X–Y–Z values calculated for HQ_1_ = 120[m^3^ s^−1^] and HQ_bf_ = 280[m^3^ s^−1^].

Scenario	Peißenberg			Unterhausen		
	X	Y	Z	X	Y	Z
**Q = 120 m**^**3**^** s**^**−1**^						
On-site	538.5	17.5	30.8	2,478	18.47	134
Equilibrium	468	21.11	22.1	10,333	109	95
**Q = 280 m**^**3**^** s**^**−1**^						
On-site	718	18.1	39	2,610	12.43	134
Equilibrium	897	33.3	27	20,000	174.9	115

All of the points of the upstream section at Peißenberg are found in cluster (2). While the on-site parameters lead to results close to the threshold between straight and alternating bars, the regime width parameters lead to the result of an alternating bar river type. The diagram shows that even though the river is channelized in this area, the results for the equilibrium width and the on-site riverbed are similar.

In cluster (3) the results for the equilibrium width river for the section in Unterhausen are found in the braided river area. As opposed to the section close to Peißenberg, in Unterhausen the river types are different between the on-site situation and the forecast for the river type occurring in case of allowed erosion processes and unsecured riverbanks. While the results for the on-site parameters show a straight single channel form, the pattern for a river with equilibrium width shows a river form with a braided multichannel riverbed. For discharges higher than HQ_1,_ it demonstrates that the braided river system will be formed. At HQ_1_ or below, the probability for braiding systems is high, since the result for the river type at this discharge is at the threshold between alternating benches and braided system.

**Figure 8:**
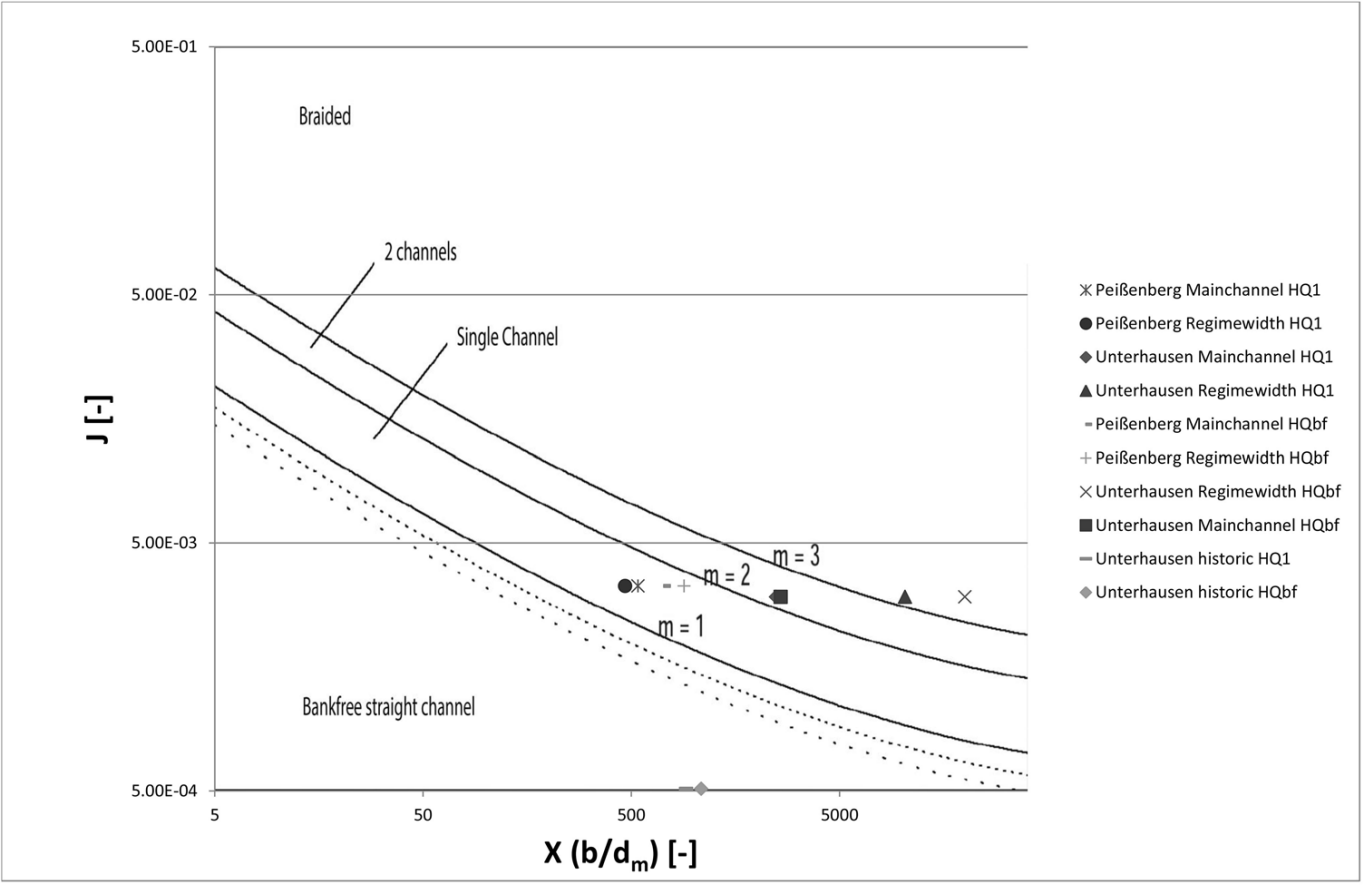
Diagram after Jäggi for in-stream morphology including results of calculations for Peißenberg and Unterhausen main channel, and equilibrium width.

Similar to the morphological classification after DaSilva, the results for the in-bed structure analysis after Jäggi (Fig. 8) shows a single-channel with bank-forming processes for the Peißenberg section. This is valid for the actual situation given as well as for the forecast for a channel with equilibrium width. For both discharges, the results show great correlation with each other. The results for the main channel are on the border between a single thread riverbed and a riverbed with two active channels. This means that the channelled river shows a tendency to in-stream bars and islands. For the regime-width calculated, the results from DaSilva/Zarn are confirmed. While the channel structure for the bankfull discharge predicts a braided/multi-channel riverbed, the lower discharges (HQ_1_) are on the threshold level between the two channel system (river with alternating bars/islands) and a braided river area. For the historic river, the diagram shows a river with a single channel and no mentionable bars. The main factor for achieving this result is the low slope, whereas for the steeper sections, a variation in X has more impact on the result.

The results show that the calculated stream power for the sections and for the historical river reached only a maximum value of 625[W m^−1^] for the section north of Weilheim to Lake Ammersee, while the channelized river has now a maximum stream power of 9211[W m^−1^] at Peißenberg section and 8320[W m^−1^] at the area of Unterhausen. For the sector at Peißenberg, however, the stream power remained the same as if there had been no major changes in the river course. Thus, the stream power in the area of Unterhausen is now 13.3 times higher than it was historically, due to the increase in slope and discharge capacity of the river channel.

### Bed material

**Figure 9:**
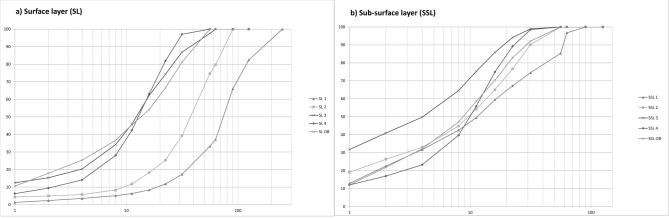
Grain size distribution along the research site at Ammer River (Bavaria/Germany) sampling sides (n = 5) from upstream (SL 1/SSL 1) to downstream (SL 4/SSL 4) and from the oxbow at Unterhausen (SL OB/SSL OB); x-axis shows grain size in [mm] y-axis the percentage in the samples.

The sediment samples from the main channel showed a downstream fining of grain size related to bed stability, which allows the assumption of a continuing transport (Fig. 9). Moreover, the degradation analysis showed that even though side erosion is prevented, the riverbed remained on a stable level over recent years, meaning that a balance in sediment supply and transport is given even without the additional input of side erosion.

### Hydrology

**Figure 10:**
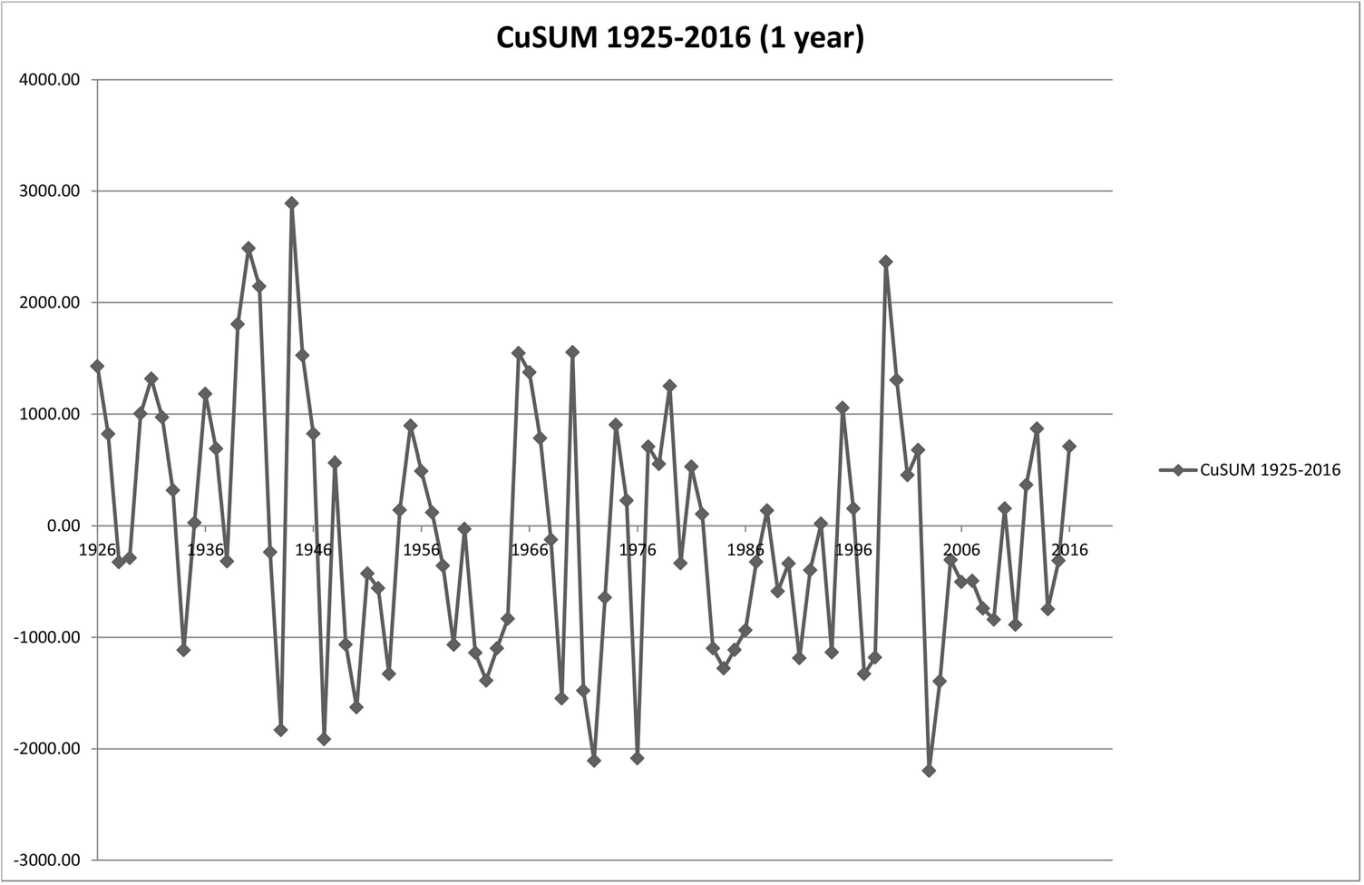
CuSUM yearly average, standardized to the yearly average the discharge series analysed showing no trend in long-term trend in discharge for the Ammer River catchment.

The discharge values since 1926 vary between 2.6 and 535[m^2^ s^−1^] as maximum daily average (peak discharge was 649[m^2^ s^−1^]). The average discharge used as constant value for c (Eq. 11) was calculated as 15.51[m^2^ s^−1^]. The cumulative deviations from the mean for the seasonal patterns also vary on an almost yearly basis in discharges and show no definite trend (Fig. 10).

## Discussion

River restoration is a highly complex field for planners, which is shown by the high rate of failure in projects throughout the world^10,26^. For restoring self-forming processes of a river, however, available space is the main criteria^83^. In literature and governmental guidelines, various ways are found to determine the corridor which is necessary for a river to be able to develop a natural morpho-dynamic status^6,34,46^. While Dahm et al.^46^ in Germany determines the development corridor based on the present (mostly anthropogenic changed) river width and factors depending on the geological and geographical area, the French approach with the “espace de liberté”^34^ is based on the historically active channel and riparian area of the river, the width of the active riverbed and the determination of a minimum functional corridor. Dahm et al.^46^ also shows a high variation between the minimum value of 320[m] and the maximum of 800[m] width for the corridor.

While the “espace de liberté” shows a variation in spatial distribution according to the historical shape, the functional corridor after Dahm is calculated for the areas with similar bed width. In case of channelized rivers, as the Ammer is, this applied approach can be used for the whole reach without any differentiation along the river’s course. As can be seen from the historical corridor, in a natural state the river width changes over the length of a river course and therefore two values which are applied on the whole stretch of a reach are not representative of the natural variation in spatial demand.

Moreover, neither system takes the on-site parameters into account. Alterations in the surrounding areas or human impacts made such as (1) increase in slope by straightening or (2) an increase in stream power due to higher maximum discharge are not considered. Even though a river needs space to develop, in settlement areas and areas with intensive agricultural use, available areas are scarce^13,84^ and former riparian areas are under human use^37,84^. Due to this limitation, the calculation of the active channel based on the 2-D data, and the 3-D analysis of river morphology and hydraulics, was compared in the presented study. The results show that under certain circumstances (section Peißenberg) the river with its actual shape in the channelized bed is close to the morphological type based on unregulated free-flowing processes. The comparison between the actual width of the channel and the theoretical value shows that rather than the suggested minimum of 320[m] (e.g. after Dahm), a channel width of about 31[m] would be sufficient for an annual flooding (HQ_1_) to have enough space for the river to be morphologically active in a natural way. A 70[m] corridor (Ashmore formula) should provide enough space for a morphologically active system. Further downstream the situation changes slightly but it can also be seen that the equilibrium width is below the calculated width after Dahm et al.^46^. Here, it is predicted that the river would form a bed with a wetted area between 125 and 241[m] corridor width, while the results for the functional corridor is between 320 and 800[m]. However, the results have to be taken with care. There are also other factors to consider. Vegetation^85,86^ and erodible bank material^69^, which are important drivers, are not taken into account for the calculations. Eaton and Millar (2004) pointed out that rivers with erodible bank material have a strong tendency to be under-predicted by the regime theory approach. In areas with higher bank stability, the prediction is more suitable^87^.This means that if applying corridors on the river, an analysis of the erodible material in the overbank area should be done first, especially as the corridor may also be limited by constructional measurements in the overbank area.

Eaton and Millar (2004) also question the approach that predictions be made for one certain discharge value. The climate area (arid/humid) and related formative discharge (Q_bf_) can strongly vary in their re-occurrence interval^88^. There is also the given influence by climate change to discharge regimes. While changes in flow are reported throughout Europe (e.g.^89^), the CuSUM-Analysis for the Ammer catchment, however, shows no trend in either direction as shown in Fig. 9. This can be said for yearly changes in discharge as well as for seasonal discharge patterns. This can be explained by the fact that besides the large weather systems (and probably changes), each smaller catchment can have its own local water cycle^90^. Thus, it can be stated that no trend can be determined on a long-term basis using historical time series for either changes in the past, and for forecasting purposes.

As pointed out in Kleinhans and van den Berg^42^ the river morphology depends on a high number of factors and the interactions between them. Therefore, simplifications to measurable parameters have to be taken into account when it comes to determination and prediction of river patterns. The analysis with two different approaches (DaSilva and Jäggi) was used for this paper as they slightly vary in their parameters. Comparing the scenarios, all results in the diagrams show good correlation for the predictions between the approaches. For the Peißenberg section, the scenarios show that the actual situation and the equilibrium width after secondary erosion are almost the same. This means that this section is, even though highly impacted with bank stabilisation and levees, still in a near-nature status concerning riverbed morphology. In this area the geological boundaries also allow the estimation of an area with high bank stability and therefore the channel width calculated can be seen as accurate^87^.

The analysis of the actual stream power pointed out that the energy within the river at a bankfull discharge is currently 13.3 times higher than it was historically. These are important findings for the boundaries in river restoration, which can only be determined due the 3-D analysis of river bathymetry and the related flow hydraulics. These results underline the importance of the ´third dimension in river restoration´. The increase in energy is given by (1) the increase in slope from historically 0.000510[m/m] to 0.00303[m/m]—and (2) the increase in discharge from Q_bf_ = 125[m^3^ s^−1^] to Q_bf_ = 280[m^3^ s^−1^]. Here, the discharge of 280[m^3^ s^−1^] was already a reduced value as it should be comparable with the former natural state of the river. According to this data, it can be said that the energy potential in the research area in Unterhausen has changed its characteristics from a lowland river to a highly active system. Such analysis is missing or can´t be derived in a 2-D analysis. The high values of the main channel indicate that depending on bank stability, the erosion potential is also 13.3 times larger than it used to be in the historic river, and is too high for the development of a meandering system (e.g.^91^). Xu points out that the threshold level between braiding and meandering depends on stream power and D_50_ along with other factors. The historical maps show that before the straightening and regulation, the Ammer River had a strong tendency for meandering along most of its reach downstream of Weilheim (2D-analysis).

The analysis of the remaining structures in the landscape and the DEM showed the same result, but the on-site parameters lead to the result of a straight to slightly bending type confirmed by the diagram after Jäggi (3D-analysis including riverbed incision over decades). Sensitivity analyses showed that changes in D_50_ to a smaller grain size, however, are resulting in a meandering characterisation. As transport capacity is too high and hydraulic boundary conditions have been changed this is not possible. The formation of a predicted braided river reach in parts of the study sites, however, implies the availability of sediment and the continuity of the transport which applies to the research site^92–95^.

According to the aims of the WFD, it is discussed that the river should be set back to its historic “reference condition”, which would mean to revert the Ammer River back to the historic bed. Studies about the changes in river morphology on a long-term basis show that this static approach should be reconsidered as rivers are a highly dynamic system, which changes depending on the supply of water and sediment^96–98^. In this case it has been proved that partial restoration to a meandering river would not be successful due to the changes in the system. Rather than putting high effort into construction work to restore the historic situation, the chance should be taken to morphologically reactivate. As well as being more cost-effective, this would also bring an advantage to the whole ecological system. New succession areas for pioneer vegetation can be created, there may be available habitat for gravel-breeding birds above the water level and gravel spawning fish, which need highly active areas, can be improved, with new habitats created^99^. Therefore, the approach of “reconstruction of historical rivers” should be questioned and reconsidered. Morphologically-active channels lead to high habitat diversity and therefore a larger bio-diversity on one site, while also having possible positive impacts on the flood situation.

## Conclusion

Rivers in industrialized countries are under anthropogenic pressure and there is a high demand for restoration projects to reach the aims of the WFD. A high percentage of the restoration throughout the world shows little or no success from an eco-morphological point of view. According to literature, the high rate of failure is based on a shortcoming of knowledge of hydro-morphological dynamic processes and a “2-dimensional” planning. Many re-meandering projects are based on historical maps, while not taking into account that the parameters on-site have changed due to human interference. There are slope, grain size, maximum or bankfull discharge factors and therefore changes to flow depth and width of the channel and the stream power. Using the example of the Ammer River, changes in the parameters have been analysed and compared between the historical and the current situation. To derive the historical riverbed, maps from 1,840 to 2017 were geo-referenced and overlaid and additional data in the remaining sections collected. Concepts to determine the necessary corridor for morphological development for the river were compared. Systems like the “espace de liberté” from France or the development corridor from Germany are based on historical patterns and have a great variety in their values and are hard to apply in populated areas. The calculation of the regime width showed that less space for a functional riverbed would be needed and is based on the actual parameters. Therefore it is more suitable for highly impacted river systems but requires more effort on-site. Analysis of a DEM and the data collected in the main channel was used to determine the situation given, and further processed in a 1D numerical model. The comparison of the parameters showed, that for one part of the research area, parameters strongly changed and therefore the morphological type of the river changed. Especially, changes in slope, discharge capacity and stream power are crucial. While still in a near-nature state (even though regulated and channelized in the upstream part), the Ammer River would change from a single-channel meandering type to a braiding river further downstream if it were given enough space to reach equilibrium width. The example also showed that parameters can change along a river stretch and restoration concepts for one section might not be suitable for another section (especially when they change in morphological type).

These results are valid for this specific case at this one river. The methodology applied only focuses on the river morphology and does not take into account the space needed for a fully-functional riparian zone, even though the riverbed is not fully covered with water all the time and creates space for primary succession.
