# Author Correction: The third dimension in river restoration: how anthropogenic disturbance changes boundary conditions for ecological mitigation

**DOI:** 10.1038/s41598-021-82524-6

**Published:** 2021-03-11

**Authors:** Martin Guzelj, Christoph Hauer, Gregory Egger

**Affiliations:** 1 Department of Water, Atmosphere and Environment, Institute of Water Management, Hydrology and Hydraulic Engineering, BOKU, University of Natural Resources and Applied Life Sciences Vienna, Vienna, Austria; 2 Christian Doppler Laboratory for Sediment Research and Management, Institute of Water Management, Hydrology and Hydraulic Engineering, BOKU, University of Natural Resources and Applied Life Sciences Vienna, Vienna, Austria; 3 Institute of Geography and Geoecology, Karlsruhe Institute of Technology, Karlsruhe, Germany

Correction to: *Scientific Reports* https://doi.org/10.1038/s41598-020-69796-0, published online 04 August 2020

This Article contains an error in the legend of Figure 1, where

“Location of the Ammer River catchment with research area marked and the channelized river-section indicated by levees along the research section on Ammer River (Sources:^57^, edited with ArcMap 10.4^58^).”

should read:

“Location of the Ammer River catchment with research area marked and the channelized river-section indicated by levees along the research section on Ammer River (Sources:^54,57^, edited with ArcMap 10.4^58^).

